# Improving Sensitive Skin Diagnosis by Integrating Diagnostic Questionnaires, Lactic Acid Sting Test, and Lipid Profiling

**DOI:** 10.1111/jocd.70099

**Published:** 2025-03-03

**Authors:** Seoyoung Kim, Kyung‐Mi Joo, Mihyun Oh, Susun An, Jieun Han, Sodam Park, Ilyoung Kwak, Dong Hun Lee, Jae Youl Cho

**Affiliations:** ^1^ Amorepacific Corporation R&I Center Yongin Korea; ^2^ Department of Integrative Biotechnology Sungkyunkwan University Suwon Korea; ^3^ Amorepacific Corporation Shanghai R&I Center Shanghai China; ^4^ Department of Dermatology Seoul National University College of Medicine Seoul Korea

**Keywords:** ceramide NPs, lactic acid sting test (LAST), questionnaire, sensitive skin

## Abstract

**Background:**

Sensitive skin (SS) is characterized by subjective symptoms, including burning, stinging, and itching, which occur with the use of cosmetics. Over 40% of the population experiences skin sensitivity, yet no clear standards for evaluating SS have been established.

**Aim:**

To diagnose SS by combining lactic acid sting test (LAST), skin irritation tests, and biophysical measurements with a developed questionnaire, validating the characteristics through quantitative analysis of natural moisturizing factors (NMF) and lipid profiles.

**Methods:**

The diagnostic questionnaires were administered to 975 healthy women from Beijing and Shanghai to evaluate their skin sensitivity. Among these, 154 participants from Beijing and 153 from Shanghai underwent physiological testing, which included a patch test, LAST, and biophysical assessments. For stratum corneum (SC) sampling, D‐squame tape was used, and the levels of NMFs and lipids were quantitatively analyzed using UPLC‐MS/MS.

**Results:**

The diagnostic questionnaires, especially when combined with LAST, improved sensitivity and reduced false negatives for identifying SS. The SS group exhibited notable differences compared to the NS group, including higher hydration and lower pH on the forehead, reduced ceramide and fatty acid levels, and fewer amino acids in the stratum corneum, although skin irritation scores were not significantly different.

**Conclusions:**

The combination of our diagnostic questionnaire with LAST was found to effectively distinguish key characteristics of SS. This methodology offers a valuable approach for enhancing the diagnosis and assessment of SS, which could, in turn, aid in the development of more targeted products for SS.

## 1 Introduction

Sensitive skin (SS) is associated with unpleasant subjective sensory reactions such as burning, stinging, and/or itching after the use of cosmetics, soaps, and other skin products. Although these unpleasant sensations cannot be explained by any skin disease, they may be accompanied by erythema [1, 2, 3]. Various attempts have been made to define SS by measuring skin susceptibility to cosmetic ingredients, such as irritants or sensory‐inducing chemicals. However, there is no international consensus on the definition and classification of SS [4, 5].

The SS is typically diagnosed through a combination of various clinical evaluations and self‐assessment questionnaires [6, 7]. The lactic acid stinging test (LAST) is frequently used to identify SS based on skin sensory irritation; however, its subjective nature may introduce intra‐individual variability [8, 9]. Furthermore, known irritants are employed to assess objective signs of SS; several studies have demonstrated that the sensitivity of one substance does not necessarily predict the sensitivity of another [10, 11, 12]. Quantitative measurements, such as transepidermal water loss (TEWL) and pH, can also be used to evaluate physiological skin changes associated with SS [12, 13, 14].

The stratum corneum (SC) is the major epidermal barrier involved in several barrier functions. Natural moisturizing factor (NMF) is a hydrophilic component consisting of amino acids and their metabolites. Derived from filaggrin, NMF is important for maintaining water balance within the SC [15, 16]. The role of the skin barrier is highly dependent on lipid organization and composition, consisting mainly of ceramides (50%), cholesterol (25%), and fatty acids (15%) [17, 18]. Skin barrier disruptions, such as atopic dermatitis, are associated with altered ceramide profiles, including reduced ceramide levels. Under these conditions, irritating substances can easily penetrate the SC and elicit skin reactions [19, 20, 21].

Several studies have been conducted to identify and evaluate SS; however, no clear standards or definitions have been established [22]. This study aims to identify an accurate diagnosis of SS by conducting LAST, skin irritation tests, and biophysical measurements in conjunction with a diagnostic questionnaire. Furthermore, the characteristics of the selected SS and non‐sensitive skin (NS) were further validated through quantitative analysis of NMF (Natural Moisturizing Factor) and lipid profiles.

## 2 Materials and Methods

### 2.1 Study Population

The study recruited 975 healthy women (575 from Beijing and 400 from Shanghai) to participate in a diagnostic questionnaire survey. Of 975 participants, 154 from Beijing and 153 from Shanghai were selected for clinical evaluations. The main inclusion criteria were as follows: (1) healthy female volunteers aged at least 18 years and (2) living in their native country for at least 5 years.

### 2.2 Test Materials

Sodium lauryl sulfate (SLS), retinol, 1,3‐butylene glycol, lactic acid, and glycolic acid were purchased from Sigma‐Aldrich (St. Louis, MO, USA) for patch testing. Other materials used in this study were of cosmetic grade and are listed in Table SI. For the sting test, 5% (w/v) lactic acid was prepared in distilled water (DW), using DW as a negative control.

### 2.3 Diagnostic Questionnaire

All the participants completed a diagnostic questionnaire assessing their general skin status, experience with cosmetic use, innate skin characteristics, environmental effects, and habits (Table SII).

### 2.4 Skin Irritation Test (Patch Testing)

Test materials (20 μL) were applied to Hayes test chambers (Hayes Service BV, Netherlands). The patches were attached to the backs of the participants for 24 h. Primary cutaneous irritation was evaluated 30 min and 1 day after patch removal. An experienced evaluator visually scored cutaneous irritation using a numerical erythema scale of 0–4.

### 2.5 The Lactic Acid Stinging Test (LAST)

The participants rested in an environmentally controlled room (temperature: 24 ± 4°C; relative humidity: 40%–45%) for 10 min after cleaning their faces with water. A cotton pad (1.5 mL/4.5 × 5.5 cm) soaked in 5% lactic acid solution was applied to the participants' cheeks, while the negative control (DW) was applied to the other cheek at the same time. Stinging and burning reactions were recorded at 10 s and every 60 s for 9 min using a scale between 0 and 3 (0 = none; 1 = slight; 2 = moderate; and 3 = severe). If the average reaction score for stinging and burning at these time points was ≥ 0.4, the participant was classified as a stinger. Participants with an average response < 0.4 were classified as non‐stingers.

### 2.6 Biophysical Measurements

Participants relaxed in a room maintained at the temperature and relative humidity described above for 10 min after washing their faces with a water. Skin sites, including the forehead and right cheek, were tested for sebum secretion (Sebumeter SM 815, Courage + Khazaka Electronic, Köln, Germany), water content (Corneometer CM 825, Courage + Khazaka Electronic), pH value (Skin‐pH‐meter pH 905, Courage + Khazaka Electronic), and TEWL value (VapoMeter SWL‐4001, Delfin Technologies, Finland).

### 2.7 Tape Stripping

Ten consecutive SC samples were collected from the cheek surface using D‐squame standard sampling discs (CuDerm Corp., Dallas, TX, USA).

### 2.8 Quantitative Analysis of NMFs in the SC

Consecutive D‐squame samples were cut and sonicated for 1 h using 1% (w/v) SLS and 2% (w/v) propylene glycol in phosphate‐buffered saline. The concentration of soluble proteins was assayed using the Pierce BCA Protein Assay Kit (Thermo Fisher Scientific, Waltham, MA, USA), according to the manufacturer's instructions. Four metabolites (pyroglutamic acid, cis‐urocanic acid, t‐urocanic acid, and citrulline) and 18 amino acids were analyzed using ultra‐performance liquid chromatography with tandem‐mass spectrometry (UPLC‐MS/MS) (Xevo TQ‐S, Waters Corp., Milford, MA, USA) and UPLC with photodiode array (ACQUITY UPLC PDA Detector, Waters Corp.).

### 2.9 Quantitative Analysis of Lipids in the SC

Authentic standards for α‐hydroxy fatty acids conjugated to phytosphingosine (CER‐AP), non‐hydroxy fatty acids conjugated to phytosphingosine (CER‐NP), non‐hydroxy fatty acids conjugated to dihydrosphingosine (CER‐NDS), non‐hydroxy fatty acids conjugated to sphingosine (CER‐NS), and saturated and unsaturated free fatty acids were used for analysis (Sigma‐Aldrich; Avanti Polar Lipids Inc., Alabaster, AL, USA; Evonik Industries AG, Birmingham, AL, USA). After sonicating the D‐squame samples for 15 min in a 2:1 solution of methanol: ethyl acetate, the extracts were filtered and dried. Dried lipids were dissolved in a 2:1 solution of chloroform: methanol, and ceramides, cholesterol, and fatty acids were analyzed using UPLC‐MS/MS.

### 2.10 Statistical Analysis

Statistical analyses were performed using the IBM SPSS software for Windows (Armonk, NY, USA). The homogeneity of variances and normality of distributions were determined using the Kolmogorov–Smirnov test. The Student's *t*‐tests, Mann–Whitney U test, and Pearson's correlation coefficient were used to assess the differences between SS and NS groups. Statistical significance was set at *p* < 0.05, and *r* values > 0.7 were considered to represent strong relationships. Additionally, SIMCA 13.0 (Umeå, Sweden) was used for Orthogonal Partial Least Squares Discriminant Analysis (OPLS‐DA).

## 3 Results

### 3.1 Sensitive Skin Ratio Compared With the Diagnostic Questionnaire and Yes/No Questions in Beijing and Shanghai

A total of 975 participants completed the diagnostic questionnaire, with 15% diagnosed with SS, and the sensitivity rate in Beijing and Shanghai was similar (Table 1). Among the participants from Beijing, a total of 154 completed the LAST, resulting in 129 non‐stingers and 25 stingers. In the stinger group, four respondents were classified as having SS based on a self‐declared YES/NO question, whereas eight participants had SS based on a diagnostic questionnaire (Table 2). This suggests that the combination of the diagnostic questionnaire and LAST may provide enhanced predictive value, with improved sensitivity and a reduced false‐negative rate.

**TABLE 1:** Result of sensitive skin ratio using diagnostic questionnaire in Beijing and Shanghai.

	Ratio of sensitive skin (% [*N*])	
	Beijing (*N* = 575)	Shanghai (*N* = 400)
Non‐sensitive	84.3 (485)	85.0 (337)
Slightly sensitive	14.3 (82)	11.0 (41)
Moderately sensitive	0.9 (5)	2.0 (14)
Strongly sensitive	0.5 (3)	2.0 (8)

**TABLE 2:** Accuracy of self‐declared YES/NO questions vs. diagnostic questionnaires in Beijing.

		Skin sensory irritation (*N* [Mean ± SE])		Number of participants
		Stinger	Non‐stinger	
Self‐declared YES/NO question	YES	4 (0.49 ± 0.097)	50 (0.08 ± 0.013)	54
	NO	21 (0.54 ± 0.062)	79 (0.06 ± 0.010)	100
Diagnostic questionnaire	Slightly to strong sensitive skin	8 (0.63 ± 0.12)	50 (0.07 ± 0.083)	58
	Non‐sensitive skin	17 (0.50 ± 0.058)	79 (0.07 ± 0.011)	96

	Self‐declared YES/NO question (%)	Diagnostic questionnaire (%)
Sensitivity	16	32
Specificity	61	61
Positive predictive value	7	14
Negative predictive value	79	82
False negative	84	68
False positive	39	39
Accuracy	54	56

Abbreviation: SE, standard error.

### 3.2 Diagnostic Questionnaire and Its Relationship With Skin Sensory Irritation and Skin Irritation

**FIGURE 1:**
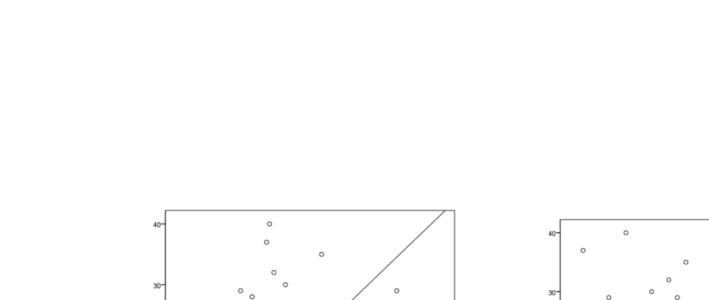
Pearson's correlation analysis among the questionnaire, sting test, and irritation test (*N* = 106). Forty‐eight questions and sensory skin irritation (*r* = 0.722, *p* < 0.01) and skin irritation (*r* = 0.221, *p* < 0.05) showed significant positive correlations between the SS and NS groups. NS, non‐sensitive skin; SS, sensitive skin.

Participants were categorized as SS and NS based on the diagnostic questionnaire and LAST assessment. Those classified as sensitive in the questionnaire and with an average score of 0.4 or higher for stinging and burning in the LAST were categorized as having SS, while those classified as non‐sensitive in the questionnaire and with a score of 0.4 or lower in the LAST were categorized as having NS. As a result, SS showed significant differences from NS in the 48 questions related to sensitive skin characteristics, including cosmetic use, innate skin characteristics, environmental impact on skin, and living habits in the diagnostic questionnaire. Although the skin irritation scores for cosmetic ingredients differed between the SS and NS groups, the differences were not statistically significant (Table 3). However, a significant trend was observed for certain cosmetic ingredients, such as pentylene glycol (*p* = 0.007), retinol (*p* = 0.052), and glycolic acid (*p* = 0.062) (Table SIII). Despite using the same classification criteria for both SS and NS, a greater number of responses in the diagnostic questionnaire, along with higher scores for both skin sensory irritation and skin irritation, were observed in Shanghai compared to Beijing (Table 3). Furthermore, a significant positive correlation was observed between the 48 questions and skin sensory irritation (*r* = 0.722; *p* < 0.01) and skin irritation (*r* = 0.221; *p* < 0.05) in both the SS and NS groups (Table 4 and Figure 1).

**TABLE 3:** Questionnaires, skin sensory irritation, and skin irritation in sensitive and non‐sensitive skin.

		Beijing (*N* = 49, S = 24, NS = 25)			Shanghai (*N* = 57, S = 28, NS = 29)		
		Mean ± SE	Mean Diff, *t*‐test/M–W		Mean ± SE	Mean Diff, *t*‐test/M–W	
			*t*/*Z* **	*p*		*t*/*Z* **	*p*
48 questions^a^ responses	SS	15.83 ± 0.91	**−**6.062**	< 0.001	23.07 ± 1.34	14.687	< 0.001
	NS	0.76 ± 0.16			2.83 ± 0.32		
Skin sensory irritation	SS	0.40 ± 0.06	**−**6.134**	< 0.001	0.6 ± 0.05	**−**6.627**	< 0.001
	NS	0.01 ± 0.00			0.01 ± 0.01		
Skin irritation	SS	3.63 ± 0.58	**−**1.200**	0.23	10.61 ± 1.33	**−**2.344**	0.019
	NS	5.28 ± 0.90			6.55 ± 0.82		

Abbreviations: NS, non‐sensitive skin; SE, standard error; SS, sensitive skin; *t*, *t*‐test.

^a^ 48 questions consisted of questions on cosmetic use (15 questions), innate skin characteristics (14 questions), environments (9 questions), and Living habits (10 questions).

** *Z* Mann–Whitney *U* test.

**TABLE 4:** Pearson's correlation analysis among questionnaire, skin sensory irritation, and skin irritation test (*N* = 106).

	48 questions responses	Skin sensory irritation	Skin irritation
48 questions responses	1		
Skin sensory irritation	0.722**	1	
Skin irritation	0.221*	0.171	1

* Correlation is significant at the 0.05 level (two‐tailed).

** Correlation is significant at the 0.01 level (two‐tailed).

### 3.3 Physiological Differences Between Sensitive and NS

We measured the values of four skin parameters (hydration, TEWL, sebum, and pH) in the forehead and right cheek. Significant differences were observed only in the hydration and pH of the forehead skin of the participants from Shanghai. The SS group had higher hydration and lower pH, respectively (Table 5).

**TABLE 5:** Biophysical measurement of sensitive and non‐sensitive skin.

		Beijing (*N* = 49, S = 24, NS = 25)			Shanghai (*N* = 57, S = 28, NS = 29)		
		Mean ± SE^a^	Mean Diff, *t*‐test/M–W		Mean ± SE	Mean Diff, *t*‐test/M–W	
			*t*/*Z* **	*p*		*t*/*Z* **	*p*
**Forehead**							
Hydration	SS	52.08 ± 3.29	**−**1.044	0.302	60.02 ± 5.45	**−**2.331**	0.020
	NS	56.34 ± 2.46			45.74 ± 2.52		
TEWL	SS	18.59 ± 1	0.448	0.656	17.55 ± 0.95	**−**0.391**	0.696
	NS	17.95 ± 1.02			16.91 ± 0.81		
Sebum	SS	53.9 ± 11.7	**−**0.300**	0.976	14.39 ± 3.55	**−**1.168**	0.243
	NS	65.1 ± 14.9			8.97 ± 1.72		
pH	SS	5.1 ± 0.11	0.840	0.405	5.18 ± 0.11	**−**0.234	0.023
	NS	4.95 ± 0.12			5.55 ± 0.12		
**Cheek**							
Hydration	SS	42.34 ± 3.53	**−**1.118	0.269	46.45 ± 2.44	0.874	0.386
	NS	47.71 ± 3.26			43.55 ± 2.27		
TEWL	SS	19.51 ± 1.2	0.923	0.361	23.37 ± 2.14	**−**1.948**	0.051
	NS	18.07 ± 0.99			18.24 ± 0.88		
Sebum	SS	63.2 ± 16.5	**−**0.51**	0.610	7.18 ± 1.58	**−**0.538**	0.591
	NS	67.6 ± 15.7			4.69 ± 0.86		
pH	SS	5.16 ± 0.09	1.744	0.088	5.47 ± 0.11	**−**1.465**	0.143
	NS	4.83 ± 0.16			5.74 ± 0.09		

Abbreviations: NS, non‐sensitive skin; SE, standard error; SS, sensitive skin; t, t‐test; TEWL, transepidermal water loss.

^a^ We calculated the mean value of three repeated measurements, except for Sebum.

** *Z*, Mann–Whitney *U* test.

### 3.4 Comparison of SC Lipid and NMF Compositions Between Sensitive and NS

Using noninvasive tape stripping, we analyzed the profiles of bioactive components in the SC, including amino acids, metabolites, and lipids (ceramides, cholesterol, and fatty acids). Total ceramide content in the SS group was significantly lower than that in the NS group, particularly in the CER NP (C16, C18, C22, C26, C28, and C30) and AP (C20, C24, and C26) series (Table 6). Furthermore, the total fatty acid content in the SS group was significantly lower in participants from Beijing. Although total NMF did not differ significantly between the SS and NS groups, the SS group had fewer amino acids and amino acid derivatives than the NS group (Table 7).

**TABLE 6:** Comparison of SC lipid composition of sensitive and non‐sensitive skin in Beijing and Shanghai.

	Beijing (mean ± SE)		Shanghai (mean ± SE)	
	Sensitive	Non‐sensitive	Sensitive	Non‐sensitive
**CER‐NP (ng/mg protein)**				
C16_NP	29.45 ± 3.39^c^	47.36 ± 4.71	5.31 ± 0.34^a^	7.87 ± 0.65
C18_NP	3.23 ± 0.35^c^	5.43 ± 0.56	5.58 ± 0.52^d^	7.63 ± 0.81
C20_NP	N.T.	N.T.	6.42 ± 0.51^a^	9.94 ± 0.98
C22_NP	1.66 ± 0.18^c^	2.29 ± 0.31	18.46 ± 1.50^a^	32.66 ± 3.40
C24_NP	5.79 ± 0.57^c^	9.5 ± 1.30	85.15 ± 7.08	148.6 ± 15.9
C26_NP	0.62 ± 0.08^c^	0.92 ± 0.13	75.97 ± 6.54^c^	145.5 ± 16.1
C28_NP	0.53 ± 0.06^d^	0.76 ± 0.11	101.54 ± 8.80^d^	160.4 ± 19.9
C30_NP	0.14 ± 0.02^b^	0.2 ± 0.02	62.82 ± 6.04^d^	98.5 ± 12.5
C32_NP	0.03 ± 0.01^c^	0.05 ± 0.01	25.88 ± 2.59	38.92 ± 5.61
Total CER‐ NP	41.44 ± 4.33^c^	66.5 ± 6.66	387.1 ± 32.1^c^	650.1 ± 72.5
**CER‐AP (ng/mg protein)**				
C16_AP	25.84 ± 2.75^d^	38.51 ± 4.30	5.31 ± 0.43	7.36 ± 0.85
C18_AP	6.79 ± 0.77^a^	12.56 ± 1.33	5.31 ± 0.43	6.59 ± 0.94
C20_AP	0.26 ± 0.05^a^	0.54 ± 0.08	5.93 ± 0.48^a^	9.89 ± 1.19
C22_AP	0.19 ± 0.02	0.24 ± 0.04	13.52 ± 1.14^c^	21.51 ± 2.44
C24_AP	6.33 ± 0.55^c^	8.98 ± 1.20	65.92 ± 5.18^a^	110.1 ± 14.1
C26_AP	1.51 ± 0.28^c^	2.6 ± 0.37	54.26 ± 4.65^c^	91.3 ± 13.2
C28_AP	0.29 ± 0.04^a^	0.47 ± 0.05	46.01 ± 4.31	73.2 ± 13.1
C30_AP	0.2 ± 0.02	0.23 ± 0.02	17.19 ± 1.78	26.75 ± 5.67
C32_AP	N.T.	N.T.	2.05 ± 0.21	3.41 ± 0.75
Total CER‐AP	41.39 ± 3.93^c^	64.13 ± 6.84	215.5 ± 17.6^d^	350.2 ± 50.8
**CER‐AS (ng/mg protein)**				
C16_AS	35.74 ± 4.46d	54.39 ± 6.42	32.38 ± 2.96	37.76 ± 4.61
C18_AS	0.27 ± 0.05d	0.47 ± 0.06	2.22 ± 0.22	6.78 ± 4.34
C20_AS	N.T.	N.T.	0.94 ± 0.10	1.24 ± 0.17
C22_AS	0.62 ± 0.09^d^	0.86 ± 0.11	2.82 ± 0.39^d^	4.09 ± 0.45
C24_AS	N.T.	N.T.	79.84 ± 8.10	99.8 ± 11.4
C26_AS	0.08 ± 0.02	0.09 ± 0.02	82.24 ± 8.64	101.7 ± 12.6
C28_AS	N.T.	N.T.	3.73 ± 0.36	4.97 ± 0.76
Total CER‐AS	37.71 ± 4.58^d^	55.8 ± 6.55	204.2 ± 20.0	256.3 ± 28.9
**CER‐NDS (ng/mg protein)**				
C16_NDS	2.98 ± 0.34^c^	5.73 ± 0.80	5.34 ± 0.51	3.99 ± 0.33
C18_NDS	0.05 ± 0.01	0.1 ± 0.04	4.54 ± 0.87	3.52 ± 0.45
C20_NDS	0.21 ± 0.02	0.21 ± 0.03	7.9 ± 0.66	7.18 ± 0.53
C22_NDS	0.64 ± 0.10	0.86 ± 0.10	20.58 ± 1.60	19.97 ± 1.59
C24_NDS	0.1 ± 0.01	0.11 ± 0.02	25.56 ± 2.12	24.07 ± 1.89
C26_NDS	0.15 ± 0.02	0.15 ± 0.02	31.52 ± 2.69	28.4 ± 2.25
C28_NDS	N.T.	N.T.	31.5 ± 2.90	30.04 ± 2.67
C30_NDS	N.T.	N.T.	20.15 ± 1.94	19.69 ± 1.83
Total CER‐NDS	4.12 ± 0.47^c^	7.15 ± 0.95	147.1 ± 11.9	136.9 ± 11.0
**CER‐NS (ng/mg protein)**				
C16_NS	24.97 ± 3.97^d^	46.8 ± 8.51	33.41 ± 4.58^d^	22.21 ± 2.48
C18_NS	0.2 ± 0.05	0.38 ± 0.07	5.74 ± 0.58	5.17 ± 0.45
C20_NS	N.T.	N.T.	2.05 ± 0.25^c^	1.27 ± 0.18
C22_NS	0.67 ± 0.09	0.79 ± 0.10	9.81 ± 1.06^d^	6.97 ± 0.74
C24_1_NS	9.95 ± 1.63^c^	16.11 ± 2.14	89.7 ± 9.61	75.3 ± 7.78
C24_NS	1.24 ± 0.14	1.64 ± 0.15	39.79 ± 3.02	34.83 ± 2.73
C26_NS	N.T.	N.T.	41.28 ± 3.33	34.07 ± 2.57
C28_NS	N.T.	N.T.	20.88 ± 1.54	19.76 ± 1.35
C30_NS	N.T.	N.T.	10.54 ± 0.75	11.25 ± 0.66
Total CER‐NS	37.04 ± 5.57^d^	65.7 ± 10.4	253.2 ± 23.0	210.8 ± 17.4
Total CER (μg/mg protein)	0.16 ± 0.01^c^	0.26 ± 0.03	1.21 ± 0.09^b^	1.6 ± 0.16
**FA (μg/mg protein)**				
C20FA	79.3 ± 7.01	94.8 ± 11.4	0.33 ± 0.03	0.38 ± 0.03
C22FA	4.62 ± 0.74^d^	5.75 ± 0.64	0.16 ± 0.01	0.19 ± 0.02
C24FA	52.46 ± 4.83^b^	75.29 ± 8.31	0.44 ± 0.04	0.58 ± 0.04
C26FA	16.79 ± 1.60^b^	23.04 ± 2.62	0.24 ± 0.02	0.32 ± 0.03
C28FA	2.04 ± 0.20^b^	3.04 ± 0.39	0.21 ± 0.02	0.31 ± 0.02
C30FA	0.69 ± 0.06^d^	0.87 ± 0.09	0.03 ± 0.00	0.04 ± 0.00
C16_1FA	202.7 ± 51.2^d^	397.5 ± 88.9	23.37 ± 3.29	30.36 ± 5.02
C18_2FA	24.86 ± 3.46	29.19 ± 4.41	1.14 ± 0.17	1.47 ± 0.20
C18_1FA	90.5 ± 12.9	127.8 ± 18.9	3.97 ± 0.42	5.13 ± 0.62
C22_1FA	0.89 ± 0.17	1.05 ± 0.15	0.05 ± 0.01	0.07 ± 0.01
C24_1FA	0.87 ± 0.19^d^	1.09 ± 0.18	0.05 ± 0.01	0.06 ± 0.01
Total FA (μg/mg protein)	475.8 ± 70.80^d^	759 ± 117	29.97 ± 3.85	38.9 ± 5.83
Cholesterol (μg/mg protein)	2.27 ± 0.32	1.71 ± 0.18	1.85 ± 0.13^b^	1.41 ± 0.13
Total lipid (μg/mg protein)	478.2 ± 70.8^b^	761 ± 117	33.02 ± 3.91	41.92 ± 5.83
CER/cholesterol (μg/mg protein)	0.11 ± 0.03^c^	0.26 ± 0.08	0.7 ± 0.05^c^	1.5 ± 0.23

*Note:* Statistical significance compared to non‐sensitive skin: ^a^ *p* < 0.01, *t*‐test; ^b^ *p* < 0.05, *t*‐test; ^c^ *p* < 0.01, Mann–Whitney *U*‐test; ^d^p < 0.05, Mann–Whitney U‐test.

Abbreviations: CER, Ceramide; CER‐AP, alpha‐hydroxy fatty acid conjμgated to phytosphingosine; CER‐AS, alpha‐hydroxy fatty acid conjμgated to sphingosine; CER‐NDS, non‐hydroxy fatty acid conjμgated to dihydrosphingosine; CER‐NP, non‐hydroxy fatty acid conjμgated to phytosphingosine; CER‐NS, non‐hydroxy fatty acid conjμgated to sphingosine; FA, fatty acids; N.T, Not tested; SC, Stratum corneum.

**TABLE 7:** Comparison of SC NMF composition of sensitive and non‐sensitive skin in Beijing and Shanghai.

	Beijing (Mean ± SE)		Shanghai (Mean ± SE)	
	Sensitive	Non‐sensitive	Sensitive	Non‐sensitive
**Amino acids (μg/mg protein)**				
His	3.04 ± 0.31	5.25 ± 1.86	2.27 ± 0.24	2.78 ± 0.41
Asn	0.54 ± 0.09	0.68 ± 0.12	0.61 ± 0.09	0.66 ± 0.11
Ser	7.41 ± 0.65	8.61 ± 1.21	3.50 ± 0.40	4.65 ± 0.84
Gln	0.93 ± 0.09	0.92 ± 0.15	1.07 ± 0.13^a^	0.53 ± 0.17
Arg	4.35 ± 0.43	4.46 ± 0.88	2.52 ± 0.23	2.82 ± 0.36
Gly	2.35 ± 0.20	2.74 ± 0.38	1.26 ± 0.14	1.62 ± 0.30
Asp	1.94 ± 0.18	2.18 ± 0.31	1.95 ± 0.18	2.30 ± 0.24
Glu	5.31 ± 0.60^b^	8.49 ± 1.26	2.86 ± 0.33	3.52 ± 0.51
Thr	2.50 ± 0.27^c^	5.43 ± 0.76	1.28 ± 0.12	1.61 ± 0.21
Ala	2.14 ± 0.22^a^	4.62 ± 0.68	0.98 ± 0.09	1.26 ± 0.22
Pro	1.27 ± 0.28^c^	3.87 ± 0.68	0.64 ± 0.05	0.76 ± 0.08
Lys	1.80 ± 0.59^c^	8.39 ± 1.56	0.84 ± 0.06	0.91 ± 0.08
Tyr	2.78 ± 0.30^c^	4.64 ± 0.44	0.62 ± 0.07	0.71 ± 0.10
Val	1.16 ± 0.09	1.28 ± 0.20	0.64 ± 0.05	0.78 ± 0.10
Leu	0.70 ± 0.07	0.79 ± 0.13	0.52 ± 0.04	0.60 ± 0.07
Ile	0.99 ± 0.10	1.06 ± 0.20	0.65 ± 0.05	0.70 ± 0.08
Phe	0.94 ± 0.08	0.92 ± 0.16	0.79 ± 0.07	0.90 ± 0.09
Trp	0.69 ± 0.06	0.74 ± 0.13	0.61 ± 0.06	0.83 ± 0.09
Total AA (μg/mg protein)	41.08 ± 3.82^d^	65.09 ± 9.13	23.61 ± 2.20	27.94 ± 3.86
**Amino acid derivatives (μg/mg protein)**				
PGA	6.03 ± 0.48	8.00 ± 1.43	4.89 ± 0.53	7.02 ± 1.34
*cis*‐UCA	0.55 ± 0.09	0.64 ± 0.16	0.33 ± 0.05	0.51 ± 0.14
*t*‐UCA	1.26 ± 0.13	1.34 ± 0.22	0.61 ± 0.10	0.69 ± 0.13
Citrulline	6.00 ± 0.51	8.95 ± 1.53	2.51 ± 0.41	3.39 ± 0.73
Total Amino Acid Derivatives (μg/mg protein)	13.83 ± 1.12	18.92 ± 2.95	8.34 ± 1.02	11.61 ± 2.31
Total NMF (μg/mg protein)	7.42 ± 1.09	16.6 ± 1.61	17.83 ± 2.59	30.16 ± 3.79

*Note:* Statistical significance compared to non‐sensitive skin: ^a^ *p* < 0.01, *t*‐test; ^b^p < 0.05, *t*‐test; ^c^ *p* < 0.01, Mann–Whitney *U*‐test; ^d^ *p* < 0.05, Mann–Whitney *U*‐test.

Abbreviations: Ala, Alanine; Arg, Arginine; Asn, Asparagine; Asp, Aspartic acid; Cis‐UCA, *cis*‐Urocanic acid; Gln, Glutamine; Glu, Glutamate; Gly, Glycine; His, Histidine; Ile, Isoleucine; Leu, Leucine; Lys, Lysine; NMF, Natural moisturizing factor; PGA, pyroglutamic acid; Phe, Phenylalanine; Pro, Proline; SE, standard error; Ser, Serine; Thr, Threonine; Trp, Tryptophan; *t*‐UCA, trans‐Urocanic acid; Tyr, Tyrosine; Val, Valine.

**FIGURE 2:**
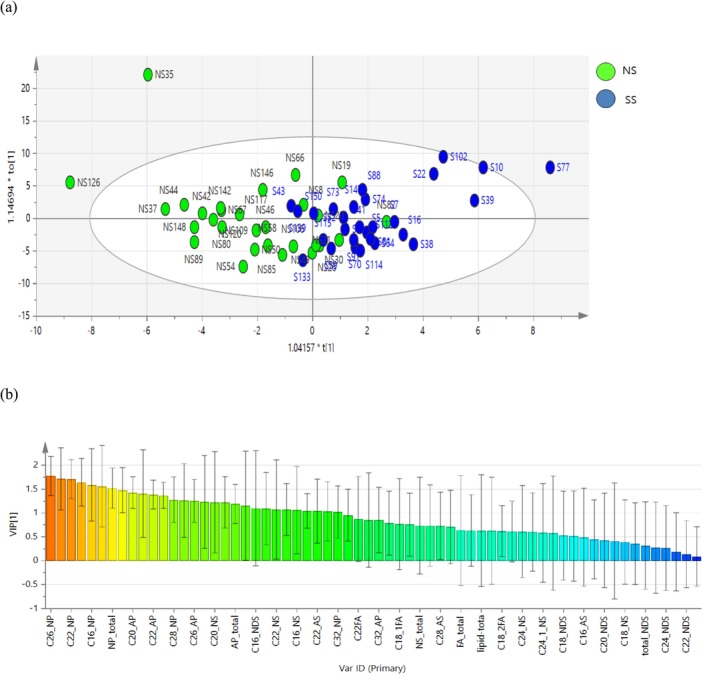
Orthogonal Partial Least Squares Discriminant Analysis (OPLS‐DA) of lipids in sensitive skin (SS) and non‐sensitive skin (NS). (a) OPLS‐DA score plots (b) Variable Importance in the Projection (VIP) scores.

Using the OPLS‐DA analysis, we found that ceramide NPs, especially C26, C22, and C16, with Variable Importance in the Projection of > 1.5, were selected as key markers relevant to the separation of these two groups (Figure 2).

## 4 Discussion

Sensitive skin is clinically characterized by sensory discomfort and may include burning, stinging, and itching, as well as clinical signs of cutaneous reactions triggered by contact with cosmetics or toiletries [5]. Recently, several studies have investigated indicators that may be useful for identifying SS [6, 23, 24, 25]; although several classifications have been suggested, there is no international consensus [26]. As the symptoms of SS are typically subjective and caused by a variety of factors, self‐declared sensitivity using questionnaires remains the most widespread means of identifying SS [6, 24]. In previous studies, approximately 40% of women in the UK and 80% in the USA reported having SS [27, 28]. Similarly, almost 40% of 9154 participants from China reported having SS [29].

Simply identifying and analyzing sensitive skin using the Yes/No method has limitations in discriminating genuinely SS. Our goal was to conduct an accurate review of products that can be safely used by individuals with highly sensitive skin. For this purpose, we developed a sophisticated diagnostic questionnaire focused on cosmetic usage to diagnose and evaluate skin sensitivity using a simple two‐step approach, combining several evaluations such as patch testing, sting tests, and physiological measurements. This integrated approach is intended to enhance the detection capabilities for skin sensitivity, thereby facilitating more precise and accurate assessments.

Our questionnaire was structured as follows: The first step involved determining the general skin status for skin discomfort after the use of cosmetics, sun sensitivity and inflammation, allergy, skin changes, and skin thickness [A]. The second step involved 48 Yes/No questions on cosmetic use, innate skin characteristics, environmental skin changes, and lifestyle habits [B]. Total scores were calculated as (2 × [A] + 3 × [B]) and were used to categorize participants into one of four groups: non‐sensitive, slightly sensitive, moderately sensitive, and highly sensitive.

As a result, the sensitivity more than doubled, and the percentage of false negatives decreased when comparing diagnostic questionnaires with self‐declared Yes/No questions. Furthermore, the use of the diagnostic questionnaire in conjunction with LAST clearly differentiated the NS group based on its characteristics. Interestingly, while a similar proportion of participants was classified as having SS in both Beijing and Shanghai, the proportion of those with moderate and strong SS was higher in Shanghai. This is likely due to the higher frequency of cosmetic use and the distinct lifestyle characteristics in Shanghai compared with Beijing [24].

Physiological differences and the composition of the SC were examined to determine whether there were differences between the characteristics of sensitive and non‐sensitive diagnosed based on our questionnaires and sting tests. According to Effendy et al. (1995), TEWL, a measure of barrier dysfunction, is positively correlated with the response to sodium lauryl sulfate [30]. However, skin hydration did not correlate well with the irritant patch test [11]. Our results showed no significant differences between SS and NS, except for hydration and skin forehead pH among participants from Shanghai. Since participants with SS are more vulnerable to barrier damage than those with NS, differences in TEWL may be observed after irritant application or desquamation [30].

Stratum corneum lipids are mainly composed of ceramides, fatty acids, and cholesterol, and their function depends on well‐organized intracellular lipids [31]. Patients with atopic dermatitis have abnormal ceramide composition in the skin caused by changes in cytokine activities in ceramide production [32]. Furthermore, our previous data showed that the amounts of skin lipid components were relatively decreased in disrupted skin with atopic dermatitis [21]. Similar to atopic skin, it was hypothesized that SS would exhibit lower lipid content compared to NS. The SS, selected according to our criteria, demonstrated a reduction in total lipid content, with ceramide levels significantly lower, particularly in the NP (C16, C18, C22, C26, C28, and C30) and AP (C20, C24, and C26) series. Furthermore, OPLS‐DA analysis identified C26, C22, and C16 from the NP series as key markers that effectively differentiate the SS and NS groups.

Combining our diagnostic questionnaire with LAST allows for a more reliable assessment of SS. Furthermore, SS exhibited a reduced total ceramide level, especially ceramide NPs, which could be a useful biomarker for SS identification. This study provides insights for improving the diagnosis and evaluation of SS, which, in turn, would help improve the accuracy of cosmetic product development for SS.

## Author Contributions

All authors have read and agreed to the published version of the manuscript.

## Ethics Statement

All participants provided their informed consent before participating in the study. The study adhered to the Declaration of Helsinki, and the study protocol was approved by the Amorepacific Institutional Review Board (IRB approval number: 2015‐1E‐N001R).

## Conflicts of Interest

The authors declare no conflicts of interest.

## Supporting information


Tables S1‐S3.

## Data Availability

The data that support the findings of this study are available on request from the corresponding author. The data are not publicly available due to privacy or ethical restrictions.
