# Supplementary material for: Improving Sensitive Skin Diagnosis by Integrating Diagnostic Questionnaires, Lactic Acid Sting Test, and Lipid Profiling
Source: J Cosmet Dermatol. 2025 Mar 3;24(3):e70099. doi: 10.1111/jocd.70099 (PMC11875041; doi:10.1111/jocd.70099)
Supplement: Supplementary file 1 — Tables S1‐S3. [file JOCD-24-e70099-s001.docx]

**Supplementary Table I.** Cosmetic ingredients used in patch test

| **INCI Name** | **CAS no.** | **Conc.** |
| --- | --- | --- |
| Sodium Lauryl Sulfate | 151-21-3 | 0.5% in DW |
| Retinol | 68-26-8 | 0.15% in 1,3-BG |
| 1,3-Butylene Glycol | 107-88-0 | 100% |
| Pentylene Glycol | 5343-92-0 | 20% in DW |
| Ethylhexylglycerin | 70445-33-9 | 0.5% in 50% 1,3-BG |
| 1,2-Hexanediol | 6920-22-5 | 20% in DW |
| Phenoxyethanol | 122-99-6 | 5% in 50% 1,3-BG |
| Octanediol | 629-41-4 | 5% in 50% 1,3-BG |
| Glyceryl Caprylate | 6015318 | 1% in 50% 1,3-BG |
| Ascorbic Acid 2-Gluoside | 6012467 | 20% in DW |
| Niacinamide | 98-92-0 | 20% in DW |
| Arbutin | 497-76-7 | 20% in DW |
| Lactic acid | 50-21-5 | 5% in DW |
| Glycolic acid | 79-14-1 | 5% in DW |

**Supplementary Table II.** Diagnostic questionnaire and judgement for assessing sensitive skin. Sensitive skin can be diagnosed (2*(A) + 3*(B)) formula. Here, A is the weighted value of General skin status and B is Cosmetic application and skin alternation. If the diagnostic value is 9 or less, it determines non-sensitive, however, over 18 categorized as severely sensitive.

| **Questionnaire** | | **Total Score** | **Weighted value** |
| --- | --- | --- | --- |
| **General skin status weighted value (A) (5-15 point)** | Experience of skin discomfort after using cosmetics, Sun sensitivity and inflammation, Allergy, Skin change, Skin thickness | 5-8 point | 1 |
|  |  | 9-10 point | 2 |
|  |  | 11-12 point | 3 |
|  |  | ≥13 point | 4 |
| **Cosmetic application and skin alternation weighted value (B) (0-48 point)** | Cosmetic uses (15 questions) | <11 point | 1 |
|  | Innate skin characteristics (14 questions) | <18 point | 2 |
|  | Environmental Skin Changes (9 questions) | <26 point | 3 |
|  | Living Habits (10 questions) | ≥26 point | 4 |

| **Estimated sensitive skin** | | | |
| --- | --- | --- | --- |
| **Sensitive skin diagnostic classification (2*(A)+3*(B))** | <10 11-14 15-17 ≥18 | GRADE I  GRADE II  GRADE III  GRADE IV | Non-sensitive Slightly sensitive Moderately sensitive Strongly sensitive |

48 questions in diagnostic questionnaire

| **Cosmetic uses** |
| --- |
| 1. After I use alcohol-rich cosmetics, my skin appears seriously stinging and burning. |
| 2. Cosmetics often cause rashes on the face. |
| 3. Use of strong fragrances may cause skin side effects. |
| 4. I often feel itchy, burning or itchy when using cosmetics. |
| 5. I can’t use cosmetics freely. |
| 6. I only use the cosmetic products that I have always used. |
| 7. There are some side effects at the beginning when changing cosmetics, but after a while it's okay. |
| 8. I use only soap I always use. |
| 9. Skin problems often occur when I change cosmetics. |
| 10. I have used cosmetics for sensitive skin. |
| 11. I have experienced side effects with sensitive skin cosmetics. |
| 12. My face has been swollen with cosmetics. |
| 13. Thick makeup can easily cause acnes on my face. |
| 14. Facial massage may cause skin side effects. |
| 15. When I remove facial mask, I might feel stinging and burning sensation. |
|  |
| **Innate skin characteristics** |
| 1. I have atopic skin. |
| 2. I had atopic skin in the childhood. |
| 3. Somebody in my family has atopic dermatitis. |
| 4. Some family members can't use any cosmetics. |
| 5. My family is generally sensitive to skin. |
| 6. I have thin skin. |
| 7. The blood vessel line can be seen on the cheek. |
| 8. The face is easy to turn red, or it is difficult to recover after then. |
| 9. I am sensitive to metal or jewelry. |
| 10. I am sensitive to pollen. |
| 11. I am sensitive to food. |
| 12. I have had skin dermatitis. |
| 13. If I have trouble on the skin, it is difficult to disappear. |
| 14. If bitten by insects, the skin swells louder than others. |
|  |
| **Environmental impact on skin** |
| 1. If I am exposed in sunshine, my face will become red, hot or itched rapidly. |
| 2. If I am exposed in cold wind, my face will turn red. |
| 3. In dust-rich area, my face will itch and produce strange stuffs. |
| 4. My skin will change based on the weather alteration. |
| 5. Temperature difference will cause the change of my face skin. |
| 6. My skin status will be influenced by the change of surrounding environment. |
| 7. My skin might become worse if water and soil alter. |
| 8. My skin seems to change along with the season. |
| 9. If I sweat, my face will itch. |
|  |
| **Living Habits** |
| 1. I have constipation. |
| 2. Often lack of sleep. |
| 3. If I have pressure, my skin will often become loose or produce acnes. |
| 4. The skin changes before and after menstruation. |
| 5. After I have spicy food, my face will produce acnes. |
| 6. My skin lacks of adopting ability. |
| 7. I take notice easily when something is growing on my face. |
| 8. I always feel cool in my hands and feet. |
| 9. Often use ointments on my face. |
| 10. I have experienced skin side effects from skin ointments. |

**Supplementary Table III.** Skin irritation score of cosmetic ingredients between sensitive and non-sensitive skin

| Ingredients |  | Shanghai (N=57, SS=28, NS =29) | | |
| --- | --- | --- | --- | --- |
|  |  | Mean ± SD | Mean Diff, M-W | |
|  |  |  | Z | p |
| Sodium Lauryl Sulfate | SS | 1.86±0.89 | -1.18 | 0.238 |
|  | NS | 1.52±0.69 |  |  |
| Retinol | SS | 1.21±1.13 | -1.94 | 0.052 |
|  | NS | 0.66±0.86 |  |  |
| 1,3-Butylene Glycol | SS | 0.36±0.68 | -1.544 | 0.122 |
|  | NS | 0.1±0.31 |  |  |
| Pentylene Glycol | SS | 0.71±0.81 | -2.716 | 0.007 |
|  | NS | 0.24±0.58 |  |  |
| Ethylhexylglycerin | SS | 0.75±0.89 | -1.658 | 0.097 |
|  | NS | 0.38±0.62 |  |  |
| 1,2-Hexanediol | SS | 0.29±0.46 | -1.263 | 0.207 |
|  | NS | 0.17±0.47 |  |  |
| Phenoxyethanol | SS | 0.5±0.79 | -0.921 | 0.357 |
|  | NS | 0.28±0.53 |  |  |
| Octanediol | SS | 0.71±0.76 | -0.783 | 0.434 |
|  | NS | 0.59±0.78 |  |  |
| Glyceryl Caprylate | SS | 0.71±0.9 | -1.54 | 0.124 |
|  | NS | 0.34±0.55 |  |  |
| Ascorbic Acid 2-Gluoside | SS | 0.29±0.6 | -0.372 | 0.710 |
|  | NS | 0.24±0.58 |  |  |
| Niacinamide | SS | 0.46±0.64 | -0.814 | 0.416 |
|  | NS | 0.31±0.47 |  |  |
| Arbutin | SS | 0.14±0.45 | -0.045 | 0.964 |
|  | NS | 0.14±0.44 |  |  |
| Lactic acid | SS | 1.21±1.1 | -1.153 | 0.120 |
|  | NS | 0.76±0.83 |  |  |
| Glycolic acid | SS | 1.39±1.17 | -1.867 | 0.062 |
|  | NS | 0.83±1 |  |  |

Z : Mann-Whitney U-test
